# Assessment of Congestion in Heart Failure Using VExUS: Current Evidence, Limitations and Clinical Perspectives

**DOI:** 10.3390/life16030518

**Published:** 2026-03-20

**Authors:** Cosmina-Georgiana Ponor, Maria-Ruxandra Cepoi, Marilena Renata Spiridon, Ionuț Tudorancea, Amelian Mădălin Bobu, Minerva Codruta Badescu, Alexandru Dan Costache, Sandu Cucută, Irina-Iuliana Costache-Enache

**Affiliations:** 1 Grigore T. Popa University of Medicine and Pharmacy, 700115 Iasi, Romania; cosmina-georgiana.ponor@d.umfiasi.ro (C.-G.P.); ionut.tudorancea@umfiasi.ro (I.T.); amelian.bobu@yahoo.com (A.M.B.); minerva.badescu@umfiasi.ro (M.C.B.); dan-alexandru.costache@umfiasi.ro (A.D.C.); cucuta.sandu@d.umfiasi.ro (S.C.); irina.costache@umfiasi.ro (I.-I.C.-E.); 2 Cardiology Clinic, “St. Spiridon” County Emergency Clinical Hospital, 700111 Iasi, Romania; marilena_spiridon@yahoo.com; 3 IIIrd Internal Medicine Clinic, “St. Spiridon” County Emergency Clinical Hospital, 700111 Iasi, Romania; 4 Cardiovascular Rehabilitation Clinic, Clinical Rehabilitation Hospital, 700661 Iasi, Romania

**Keywords:** systemic venous congestion, VExUS, heart failure, congestive organ dysfunction

## Abstract

*Background*: Systemic venous congestion is a key driver of organ dysfunction in heart failure (HF), yet accurate non-invasive quantification remains challenging. Recognizing residual congestion is critical, since it predicts HF readmissions and mortality. Traditional assessments (physical exam, jugular venous pressure, inferior vena cava [IVC] size) are imprecise. The Venous Excess Ultrasound Score (VExUS) is a semi-quantitative point-of-care ultrasound (POCUS) protocol that integrates IVC diameter with Doppler flow patterns in the hepatic, portal and intrarenal veins to grade systemic venous overload. *Methods*: We conducted a narrative review of literature (2018–2025) regarding the usefulness of VExUS in HF, covering congestion pathophysiology, clinical evidence (hemodynamic correlations, organ dysfunction, outcomes), potential applications, integration with lung ultrasound, echocardiography and biomarkers, limitations of its assessment and future directions. *Results and Discussions*: In HF, elevated right atrial pressure causes venous congestion. VExUS integrates IVC diameter with Doppler waveforms of hepatic, portal, and intrarenal veins to grade congestion. Emerging evidence shows higher VExUS grades correlate with elevated filling pressures, renal dysfunction, and worse outcomes. Its use may guide diuretic therapy, aid discharge planning, and monitor outpatient congestion, especially when combined with lung ultrasound and biomarkers. However, VExUS has limitations: it is technical and operator-dependent. Importantly, large trials validating VExUS-guided management are lacking. Future directions include AI-driven automation of Doppler analysis and integration with multimodal congestion monitoring to provide a comprehensive congestion assessment. *Conclusions*: VExUS is a promising noninvasive tool for quantifying congestion in HF. Higher grades are associated with organ dysfunction and poor prognosis. Incorporating this technique into HF care may improve congestion-guided therapy, but large-scale validation is required before routine use.

## 1. Introduction

Heart failure (HF) has become a major global public health challenge, affecting an estimated 64 million people worldwide, with an increasing prevalence driven largely by population aging [1].

Congestion represents a hallmark feature of HF, being present in more than 97% of patients with acutely decompensated HF, whereas patients with de novo HF primarily present with cardiogenic shock or acute pulmonary edema. Residual congestion, defined as elevated left ventricular diastolic pressure associated with persistent signs and symptoms of HF, such as dyspnea, pulmonary crackles and peripheral edema, despite guideline-directed medical therapy, constitutes the main risk factor for HF-related readmissions and mortality. Therefore, prompt recognition of congestion and rapid optimization of therapy are crucial to induce remission in this malignant process [2].

Physical examination alone is often unreliable in accurately assessing the degree of congestion. The incorporation of ultrasound for hemodynamic assessment may be advantageous in this setting and has become an essential bedside tool in modern clinical practice; however, it should not replace thorough medical history-taking and careful clinical examination, including assessment of capillary refill time or evaluation of the jugular veins.

Given the limitations of inferior vena cava (IVC) sonographic assessment, such as in patients with cirrhosis, physiological dilation in athletes, smaller IVC dimensions proportional to short stature, conditions associated with increased intra-abdominal pressure, or IVC dilation in intubated and mechanically ventilated patients, the VExUS (Venous Excess Ultrasound Score) protocol, a point-of-care ultrasound (POCUS) technique, has emerged as a valuable tool for the non-invasive quantification of systemic venous congestion. Elevated VExUS scores have been associated with organ dysfunction, likely due to reduced perfusion pressure in the setting of increased central venous pressure. Perfusion pressure, defined as the difference between mean arterial pressure and central venous pressure, has been shown to be associated with an increased risk of organ injury, when reduced. In addition, VExUS not only allows non-invasive quantification of venous congestion but also enables real-time assessment of dynamic changes in response to therapy [3,4,5].

The introduction of VExUS marked a major conceptual advance in the non-invasive assessment of systemic venous congestion. Initially developed in the intensive care setting, the score was proposed to overcome the limitations of traditional methods used to assess volume status, particularly the excessive reliance on inferior vena cava diameter, a marker that is often nonspecific in certain clinical contexts. VExUS integrates ultrasonographic evaluation of the inferior vena cava with Doppler analysis of hepatic, portal, and renal venous flow, thereby quantifying the impact of elevated right atrial pressure on the entire abdominal venous system. Early studies conducted in intensive care units demonstrated that elevated VExUS scores correlate with organ dysfunction, particularly postoperative renal impairment, underscoring the importance of venous congestion as a major and independent hemodynamic determinant of prognosis [6].

The objective of this narrative review is to critically analyze the existing evidence regarding the utility of VExUS in the assessment and monitoring of venous congestion in patients with HF, highlighting its advantages, limitations, and integrative potential in optimizing therapeutic decision-making.

## 2. Methods

This narrative review is designed to provide a critical and multidimensional synthesis of the current literature regarding the application of the Venous Excess Ultrasound Score (VExUS) in the assessment of systemic venous congestion among patients with HF. The methodological approach adhered to international editorial standards for scholarly narrative reviews and did not follow the prespecified selection or analytical frameworks required for systematic reviews or meta-analyses.

A formal literature search was conducted in PubMed/MEDLINE, EMBASE, and the Cochrane Library, covering the period from 1 January 2018, to 1 September 2025. The search strategy was predefined and incorporated relevant MeSH terms and keywords, including: “Venous Excess Ultrasound Score”, “VExUS”, “systemic venous congestion”, “portal vein Doppler”, “hepatic vein Doppler”, “intrarenal venous flow”, “right atrial pressure”, “venous hypertension”, “hemodynamic assessment”, “heart failure”, and “point-of-care ultrasound”. Searches were restricted to peer-reviewed articles published in English.

Eligibility was determined through title and abstract screening, followed by full-text evaluation of potentially relevant manuscripts. Included studies consisted of observational cohorts, prospective or retrospective analyses, post hoc investigations, case series, methodological reports, conceptual papers, and consensus documents that examined either the VExUS score or its individual components in the context of hemodynamic assessment in HF. Exclusion criteria encompassed experimental animal studies, research not involving ultrasound-based assessment of venous congestion, clinically irrelevant reports, and insufficiently documented publications.

To enhance completeness, reference lists of included studies were manually screened using a “snowball search” technique to identify additional pertinent articles not captured in the initial electronic search. Furthermore, contemporary international guidelines (ESC; AHA/ACC/HFSA) were reviewed for complementary evidence.

This methodological framework enables the integration of pathophysiological, diagnostic, and clinical perspectives, supporting a rigorous and comprehensive evaluation of the VExUS score and its potential role in refining the assessment of systemic venous congestion in HF care.

## 3. Pathophysiology of Venous Congestion in Heart Failure

### 3.1. Systemic Venous Hypertension and Organ Congestion

In HF, the failing heart’s reduced pumping ability leads not only to forward perfusion failure but also to backward failure, elevated systemic venous pressures that cause multiorgan congestion [7,8]. High right atrial and central venous pressures are transmited backward, overloading the venous capacitance system and leading to passive congestion of organs such as the liver, kidneys, and the splanchnic circulation [9]. Clinically, right-sided HF often manifests with abdominal congestion, including congestive hepatopathy), ascites, bowelwall edema and peripheral edema. The liver becomes enlarged and tender with a positive hepatojugular reflux, and persistent venous stasis can eventually lead to hepatic fibrosis and cardiac cirrhosis. Similarly, elevated venous pressure in the gastrointestinal tract and spleen causes splanchnic sequestration of fluid, contributing to ascites and gut edema (which can impair nutrient/drug absorption and promote cachexia). Thus, increased systemic venous pressure in HF precipitates a cascade of congestive organ dysfunction [10,11,12].

### 3.2. Renal Venous Congestion and Worsening Renal Function

Renal congestion is especially critical, as it is a major determinant of worsening renal function (WRF) in HF. Elevated central venous pressure (CVP) impedes renal venous outflow, raising renal interstitial pressure and renal vein pressure. This venous hypertension has a twofold deleterious effect on the kidneys: (1) it directly reduces the net glomerular filtration pressure by diminishing the trans-glomerular gradient (akin to a “renal tamponade” effect of pressure surrounding the nephron), and (2) it indirectly lowers renal arterial perfusion pressure by reducing forward cardiac output [13]. The result is often a drop of glomerular filtration rate and sodium/water excretion. Indeed, clinical studies have shown that high right-sided filling pressures (high CVP) correlate strongly with renal dysfunction in HF, more so than low cardiac output in many cases [14]. This mechanism, commonly referred to as “congestive nephropathy”, explains why patients with severe right-sided congestion frequently develop renal impairment. In summary, venous congestion in HF leads to renal venous hypertension and relative hypoperfusion, causing WRF and activating neurohormonal pathways (RAAS, SNS) that further exacerbate fluid retention [13]. Recognizing renal congestion as a driver of cardiorenal syndrome is important, as mere improvement of forward output may not reverse WRF unless venous pressures are relieved.

### 3.3. “Silent” Congestion—Limitations of Clinical Signs and Role of Ultrasound

A key challenge in HF management is that clinical signs of congestion are insensitive and often late indicators. By the time a patient has obvious jugular venous distension, hepatomegaly, or pitting edema, there may have been weeks of subclinical fluid accumulation. Many patients can have significant elevation of filling pressures and venous congestion without obvious outward signs or symptoms.

Given these limitations, imaging, particularly ultrasound-based, has emerged as a crucial tool for detecting “silent” congestion before it becomes clinically overt. Point-of-care ultrasound can objectively assess venous pressure and fluid overload. For example, a plethoric IVC with poor inspiratory collapse on echocardiography indicates elevated right atrial pressure even if jugular veins look clinically normal. Likewise, tissue Doppler and mitral inflow can estimate left-side filling pressures. Venous Doppler ultrasound of abdominal organs enables assessment of organ congestion. Pulsatile, reversed flow patterns in hepatic veins or portal vein on Doppler strongly suggest high right-sided pressures and systemic congestion. Abnormal renal vein flow (e.g., discontinuous or biphasic flow) is another ultrasound marker of renal congestion in HF [15]. In addition, lung ultrasound (LUS) can detect pulmonary interstitial edema via B-lines earlier than crackles are heard on auscultation. Studies have shown that subclinical pulmonary congestion (multiple B-lines on LUS) can exist in HF patients without dyspnea, and portends worse outcomes if untreated [10,16].

Integrating these imaging findings allows clinicians to unmask congestion that physical exam misses. For example, an elevated VExUS grade or a high B-line count can confirm volume overload even in patients who appear clinically euvolemic. Early detection of occult congestion by ultrasound is crucial, as it enables timely intensification of diuretic or vasodilator therapy before full decompensation occurs.

**Figure 1:**
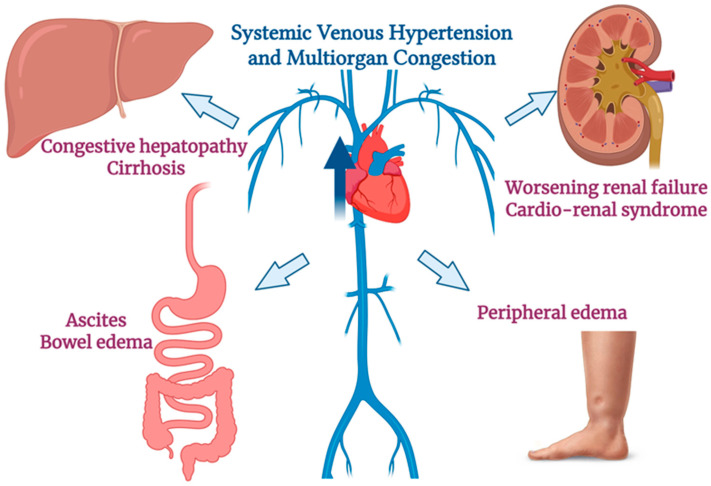
***Pathophysiology of venous congestion in heart failure***. Backward transmission of elevated right atrial and central venous pressures leads to systemic venous congestion, affecting the liver, kidneys, and splanchnic circulation and resulting in clinical manifestations such as congestive hepatopathy, cirrhosis, ascites, worsening renal failure, bowel wall edema, and peripheral edema. Created in BioRender. Ponor, C. (2026) https://BioRender.com/zojzifo (accessed on 25 January 2026).

In summary, venous congestion in HF is a central pathophysiological process that leads to hepatic, splanchnic, and renal dysfunction (Figure 1). Backward pressure on the venous system, especially when prolonged, causes organ injury (congestive hepatopathy, congestive nephropathy, etc.) and worsens outcomes. Because clinical signs often appear late or may even be absent in a significant proportion of patients, modern HF management increasingly relies on imaging modalities like ultrasound to detect and monitor “silent” congestion. This comprehensive approach may help guide therapy (e.g., adjusting diuretics) to relieve congestion in order to prevent organ damage and HF hospitalizations.

## 4. Description of the VExUS Score

VExUS combines an assessment of the IVC diameter/collapsibility with Doppler flow patterns in three organ venous systems, the hepatic veins, portal vein, and intrarenal (interlobar) veins, to quantify the severity of venous congestion [6]. This comprehensive approach addresses the limitation that a dilated IVC alone does not always equate to fluid overload (e.g., IVC diameter can be influenced by mechanical ventilation, pulmonary hypertension, chronic RV dysfunction, severe tricuspid regurgitation, high intra-abdominal pressure) [17]. It helps detect early signs of systemic venous hypertension affecting the liver, gut, and kidneys. The exam is performed in four steps, as outlined below:

(1) ***IVC Assessment***: The first step is to measure the IVC’s maximal diameter and its respiratory collapsibility (inferior vena cava collapsibility index) as an estimate of central venous pressure (RAP/CVP). An IVC diameter >~2.0 cm with poor inspiratory collapse suggests elevated right atrial pressure (e.g., an IVC > 2.1 cm with <50% collapse corresponds to a mean RAP ~10–20 mmHg). Conversely, a smaller IVC (<~2.1 cm) that collapses >50% with inspiration indicates low filling pressures (RAP ~0–5 mmHg) [17]. In the VExUS protocol, a dilated IVC ≥ 2.0 cm with minimal collapse is considered a requisite for significant congestion—if the IVC is <2 cm and collapsible, there is no significant venous congestion, the VExUS score is 0 and the exam can be stopped at this point. If the IVC is ≥2 cm, one proceeds to evaluate the abdominal organ Doppler flow patterns in the next steps.
(2) ***Hepatic Vein Doppler (Hepatic Congestion Pattern)*:** Using a subcostal view of the hepatic veins draining into the IVC, a pulsed-wave Doppler trace is obtained in a hepatic vein (commonly the right or middle hepatic vein). The normal hepatic vein waveform is dominant forward flow in systole (S-wave) that is larger than the diastolic wave (D-wave) (S > D), reflecting unhindered venous return into the right atrium during systole. As venous congestion increases (e.g., rising RA pressure or tricuspid regurgitation), the systolic forward flow diminishes. In mild hepatic congestion, the S-wave falls below the D-wave in amplitude (S < D, but still toward the heart). In severe congestion, the hepatic S-wave inverts (systolic flow reversal), meaning blood flows retrograde into the liver during RV systole. This hepatic vein Doppler progression—from S > D (normal) to S < D (mildly abnormal) to S reversal (severely abnormal)—is a key indicator of elevated right atrial pressure and venous back-up in the liver. A reversed hepatic S-wave (also called systolic flow reversal) is considered a severe Doppler abnormality in the VExUS scoring system, usually signifying advanced congestion (often seen with severe tricuspid regurgitation or right heart failure) [18].
(3) ***Portal Vein Doppler (Portal Venous Pulsatility)*:** Next, the portal vein flow is examined. Normally, portal vein flow is monophasic, with minimal pulsatility, a steady forward flow into the liver throughout the cardiac cycle. As right-sided pressures rise, the hepatic sinusoidal pressure increases and oscillations from the cardiac cycle transmit backward into the portal circulation, causing the portal flow to become pulsatile. The degree of portal flow pulsatility can be quantified by a pulsatility index: (Vmax − Vmin)/Vmax × 100. In congestion, this index rises as the trough (Vmin) drops with each right atrial contraction. Mild portal congestion is defined by a pulsatility index around 30–50%. Severe portal venous congestion is present when pulsatility exceeds 50%, meaning a highly phasic portal flow [6]. In such cases, the forward portal flow nearly ceases or even transiently reverses during systole due to the elevated venous pressure transmitted from the heart. A normal portal vein waveform (continuous, <30% pulsatility) is considered a VExUS grade 0 finding, whereas marked pulsatility ≥50% is categorized as a severe abnormality.
(4) ***Intrarenal Venous Flow (Renal Vein Doppler)*:** Finally, Doppler interrogation of an intrarenal (interlobar) vein is performed with color Doppler and pulsed-wave Doppler adjusted to locate small interlobar vessels. In healthy volume status, the intrarenal venous flow is continuous and monophasic (a steady non-pulsatile venous waveform below the baseline). With rising venous congestion, the renal vein waveform starts to oscillate in tandem with the cardiac cycle, analogous to the portal pattern. Mild renal congestion yields a pulsatile biphasic waveform with a diminished systolic forward flow and a distinct diastolic phase (often termed “renal venous pulsatility” or a biphasic discontinuous flow). In more severe congestion, the systolic component of renal venous flow disappears entirely, leaving only a diastolic-forward flow with gaps during systole. This results in a monophasic, discontinuous pattern where flow is present only in diastole and absent in systole, a severe abnormality indicating high renal vein pressures and venous stasis [19].

**Figure 2:**
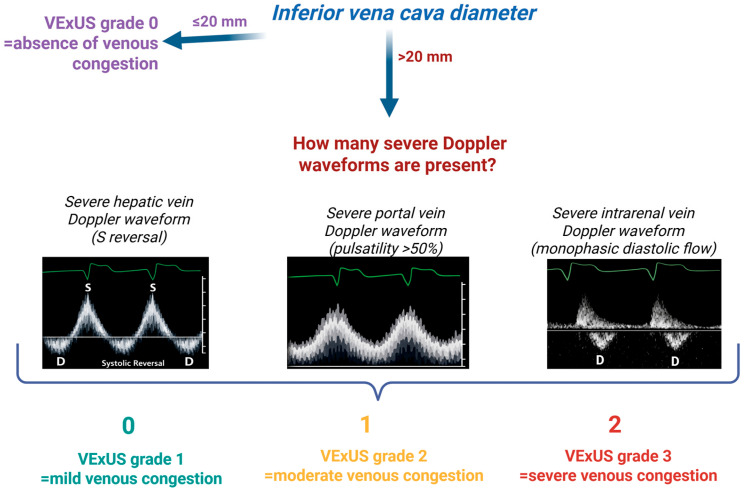
***Steps of VExUS grading.*** Schematic representations of the severe Doppler waveforms of the hepatic, portal and intrarenal veins. The first step is the measurement of the IVC (inferior vena cava) diameter. If the IVC < 20 mm, the VExUS grade is 0. If the IVC > 20 mm, the grade of the VExUS score is determined by the number of severe Doppler waveforms present in the case (zero severe Doppler waveforms = VExUS grade 1; one severe Doppler waveform = VExUS grade 2; two severe Doppler waveforms = VExUS grade 3). Created in BioRender. Ponor, C. (2026) https://BioRender.com/sfb9l2a (accessed on 13 March 2026).

After completing these four assessments, the findings are integrated into an overall VExUS grading scale from 0 to 3 (higher grades = worse congestion). The Doppler waveform in each organ system is characterized as normal, mildly abnormal, or severely abnormal as defined above. Grade 0 (no congestion) is assigned when the IVC is not dilated (<2 cm with normal collapsibility). Grade 1 (mild congestion) is defined by a dilated IVC (≥2 cm) but only normal or mild Doppler changes in the organ veins (no “severe” waveform abnormalities are present). Grade 2 (moderate congestion) indicates a dilated IVC plus one severely abnormal Doppler pattern (severe congestion in one organ system). Grade 3 (severe congestion) is defined by a dilated IVC (≥2 cm) accompanied by severe Doppler abnormalities in two or more organ systems. In practice, grade 3 presents as a plethoric IVC with at least two of these severe findings: reversed hepatic vein flow, >50% portal pulsatility, and absent systolic renal flow. This highest grade reflects advanced systemic venous congestion affecting multiple organs. The VExUS score thus provides an escalating scale (0–3) to objectively quantify congestion, with grade 3 denoting severe systemic congestion that merits prompt clinical attention and intervention [18] (Figure 2).

## 5. Results and Discussion

### 5.1. Clinical Evidence of VExUS in Heart Failure

In recent years, increasing attention has been directed toward ultrasound-based assessment of systemic venous congestion. Several observational studies conducted in critically ill and HF populations have evaluated the diagnostic, physiological, and prognostic implications of the VExUS score and its components. Key clinical studies investigating the VExUS protocol across different clinical settings, particularly in patients with HF, are summarized in Table 1, highlighting study populations, sample sizes, study design, and principal outcomes.

Collectively, these studies suggest that ultrasound markers of systemic venous congestion may provide clinically relevant information regarding hemodynamic status and prognosis. However, most available data originate from observational cohorts with relatively small sample sizes, and prospective trials evaluating VExUS-guided therapeutic strategies remain limited.

### 5.2. Intensive Care Studies That Generated the VExUS Concept

The VExUS concept was initially proposed in the intensive care setting as an integrated ultrasound-based score for quantifying systemic venous congestion. A landmark study in a cohort of 145 post–cardiac surgery patients demonstrated that severe venous congestion identified by ultrasound (defined as an inferior vena cava (IVC) diameter ≥2 cm combined with severe abnormal Doppler patterns in the hepatic, portal, and intrarenal veins) was strongly associated with the incidence of postoperative acute kidney injury (AKI). Patients presenting with a VExUS score indicative of severe congestion at ICU admission had a markedly increased risk of developing AKI (hazard ratio approximately 3.7), an association that remained statistically significant after adjustment for baseline renal risk factors and perioperative variables [6].

Moreover, the presence of severe VExUS congestion at admission provided superior predictive performance compared with traditional hemodynamic measurements. In particular, VExUS clearly outperformed CVP in identifying patients at risk for acute renal dysfunction, underscoring the clinical relevance of systemic venous congestion as a mediator of critical organ injury in critically ill patients [6]. These findings suggest that venous congestion, alongside arterial hypoperfusion, is a key pathophysiological determinant of AKI in the ICU population.

Although initially validated in the postoperative cardiac surgery setting, the broader applicability of VExUS in general intensive care populations remains under investigation. For instance, a recent prospective study in a non-cardiac ICU cohort reported a relatively low prevalence of moderate-to-severe venous congestion (VExUS grade ≥ 2 in approximately 22% of patients) and failed to demonstrate a significant association between admission VExUS scores and subsequent AKI or 28-day mortality [27]. These findings suggest that the predictive value of VExUS may be context and population-dependent, highlighting the need for further research focused on specific critically ill subgroups. Nevertheless, early ICU data, particularly from cardiac surgical populations, laid the conceptual foundation for VExUS by clearly demonstrating the link between systemic venous congestion and organ dysfunction, especially AKI, thereby opening the path for its application in other clinical contexts.

### 5.3. Evidence Supporting VExUS in Heart Failure (Pilot and Observational Studies)

Following the definition of the VExUS score in the ICU, attention shifted toward HF, a clinical syndrome in which venous congestion plays a central role in prognosis. Numerous pilot and observational studies have explored the application of VExUS in patients with acute or chronically decompensated HF, investigating its hemodynamic correlations, prognostic value, and potential role in guiding therapy. The key findings are summarized below.

#### 5.3.1. Correlation Between VExUS and Hemodynamic Parameters

One of the first research directions evaluated whether the VExUS score reflects central hemodynamic parameters in HF patients, particularly right and left sided filling pressures. Available data, although derived from relatively small cohorts, are encouraging. A good concordance has been observed between VExUS and right atrial pressure. Specifically, several studies reported a strong correlation between VExUS values and invasively measured right atrial pressure or central venous pressure, suggesting that elevated VExUS scores reliably indicate increased right-sided filling pressures [23,26,28,29].

In acute HF cohorts, higher VExUS grades have been shown to correspond to higher left-sided filling pressures. Patients with severe systemic congestion (VExUS 3) had significantly elevated echocardiographic E/e′ ratios (a surrogate for left ventricular end diastolic pressure) compared to those with lower VExUS grades. In other words, VExUS score correlated with indicators of elevated left ventricular end-diastolic pressure. In this study, mean E/e′ rose markedly in the VExUS 3 group, supporting the concept that severe multiorgan venous congestion occurs alongside increased LV filling pressures [20].

Notably, VExUS grade 3 (“severe congestion”) identifies patients with the highest left-sided filling pressures. In the same cohort, grade 3 patients had mean E/e′ ratios significantly above those in VExUS 0–2. Thus, an elevated VExUS (particularly grade 3) was tightly linked to evidence of left ventricular overload [20]. This finding (that VExUS 3 carries higher E/e′ than lower grades) underlines VExUS’s utility as a marker of left-sided congestion.

Elevated VExUS also mirrors right-heart strain. VExUS 3 patients exhibited larger right ventricular dimensions, severe tricuspid regurgitation, and impaired right atrial function (reduced RA strain) [20]. In ambulatory HFrEF patients, high VExUS scores were associated with clear signs of RV dysfunction: reduced TAPSE and RV fractional area change, severe tricuspid regurgitation, and higher estimated systolic pulmonary artery pressure [24]. Together, these studies show that systemic venous congestion on ultrasound corresponds to adverse RV remodelling and tricuspid valve regurgitation, as expected in decompensated HF.

Current literature supports the integration of multiple imaging techniques to better characterize the complex hemodynamic profile of HF. Recent work emphasizes the value of combining advanced echocardiographic techniques (three-dimensional imaging, myocardial strain analysis, and vortex flow assessment) to obtain a more comprehensive evaluation of cardiac structure, function, and intracardiac hemodynamic [30]. The integration of systemic venous Doppler evaluation could further enhance multimodal congestion assessment by extending imaging beyond the cardiac chambers to the systemic venous circulation, for a more thorough characterization of the HF phenotype.

Importantly, these findings highlight the incremental value of VExUS over isolated IVC assessment. Simple IVC diameter measurement has well-recognized limitations—such as modest correlation with right atrial pressure and susceptibility to respiratory and mechanical factors—whereas the multiorgan VExUS approach more accurately captures the global congestion burden. In a study of 290 patients with acute HF, incorporation of VExUS into a clinical model significantly improved the prediction of in-hospital mortality, whereas other congestion indices such as IVC diameter or right atrial function did not confer additional prognostic value [20].

Collectively, these data indicate that VExUS correlates well with intravascular hemodynamic status on both the right and left sides of the heart and represents a more comprehensive marker of congestion than isolated venous measurements.

#### 5.3.2. Prediction of Decompensation and Rehospitalization

A major practical challenge in HF management is identifying patients at high risk of recurrent decompensation and rehospitalization following an acute episode. In this context, investigators have explored the ability of VExUS to predict short-term outcomes after discharge. Initial evidence suggests a clear association between residual venous congestion at discharge and adverse events. In a cohort study involving 49 patients with acute decompensated HF and reduced ejection fraction, approximately one-third of patients exhibited significant systemic congestion at discharge (VExUS grade 2 or 3) despite apparent clinical stabilization. During 90-day follow-up, patients discharged with VExUS ≥ 2 had significantly higher rates of HF readmission or emergency visits compared with those with VExUS 0 (35.3% vs. 9%, *p* = 0.044) [21].

Also, a modified version (mVExUS) of the score (using only the hepatic and portal venous waveforms) showed promising results, being assessed in a dynamic manner (within 24 h of admission and repeated at 72 h). A reduction of ≥1 point in the mVExUS score over the first 72 h (ΔVExUS ≥ 1) was independently associated with lower in-hospital mortality and was accompanied by favourable clinical and laboratory markers of decongestion in a recent study [4].

These findings support the hypothesis that persistent venous congestion detected by ultrasound marks increased vulnerability to early decompensation and may identify patients requiring closer post-discharge monitoring and management. However, these studies were small and often single-center. Despite these limitations, the emerging signal is consistent: patients with elevated VExUS scores at discharge appear to face a higher risk of early HF recurrence, underscoring the potential role of VExUS in assessing residual congestion as part of discharge planning.

#### 5.3.3. Relationship Between VExUS, Renal Congestion and Worsening Renal Function

The interaction between HF and renal function (the cardiorenal syndrome) is strongly mediated by venous congestion. Contemporary studies have demonstrated that systemic venous stasis contributes substantially to worsening renal function (WRF), in some cases more than reduced cardiac output. According to the “backward failure” paradigm, elevated central and renal venous pressures impair glomerular filtration through hemodynamic and neurohormonal mechanisms. Clinical observations confirm that CVP is a major predictor of renal function decline in HF patients, independent of left ventricular systolic function [31].

In this context, VExUS, particularly its intrarenal Doppler component, provides a direct window into early renal congestion. Abnormal intrarenal venous flow patterns (discontinuous or reversed flow) have been shown to represent early markers of renal venous congestion. A prospective observational study indicates that abnormal renal Doppler patterns at admission for acute HF are significantly associated with subsequent cardiorenal events, including WRF and need for renal replacement therapy. Moreover, dynamic worsening of these Doppler patterns during hospitalization predicts particularly unfavourable outcomes [32].

VExUS can identify patients at risk before serum creatinine rises (elevated VExUS scores preceded the development of AKI in postoperative patients, confirming its predictive value) [6]. Overall, the literature underscores systemic venous congestion as a key determinant of WRF in HF, with VExUS providing a practical bedside tool to quantify this pathophysiological process. Identification of a high VExUS score could alert clinicians to impending cardiorenal syndrome and prompt early optimization of volume management to prevent progressive renal injury.

#### 5.3.4. Role of VExUS in Guiding Therapy

Given the close link between congestion and prognosis in HF, an important question is whether VExUS-guided decongestive therapy (using diuretics and vasodilators) can improve clinical outcomes. While high-quality randomized evidence is still lacking, some pilot and observational studies suggest that adjusting diuretic and vasoactive therapy based on venous Doppler patterns may facilitate more effective decongestion and improve cardiorenal surrogates [25,33,34].

In cases where VExUS remains elevated despite aggressive diuresis, persistent congestion or diuretic resistance should be suspected. Early identification of such discordance enables timely therapeutic adjustment, such as escalating diuretic doses, combining diuretic classes, or adding vasodilators, before refractory edema or renal deterioration develops. Clinical reports suggest that improvement or normalization of portal vein Doppler pulsatility during decongestive therapy may parallel resolution of venous congestion and recovery from congestion-related kidney dysfunction in selected patients [35,36]. Additionally, changes in portal venous flow have been shown to predict the ability to achieve substantial negative fluid balance (>5 L) [37].

In one study, patients presenting with higher VExUS grades (severe venous congestion) had significantly lower loop diuretic efficiency, having much less diuresis per 40 mg furosemide dose, compared to those with low congestion (VExUS 0–1). This indicates that elevated systemic venous pressure contributes to diuretic resistance. Notably, the association between high VExUS and poor diuretic response was independent of baseline kidney function, underscoring congestion (rather than initial creatinine) as a key factor in diuretic efficacy [25].

Although large randomized trials of VExUS-guided therapy are still lacking, current evidence suggests that incorporating this score into daily monitoring provides valuable feedback. VExUS may help clinicians determine whether to continue aggressive decongestion or to temper therapy to avoid hypovolemia and renal hypoperfusion. Consequently, the score holds promise as a practical tool for personalized management of decompensated HF, helping balance effective decongestion with prevention of therapy-related adverse effects. This approach aligns with emerging therapeutic frameworks that emphasize intensified or advanced pharmacological strategies for patients with persistent congestion. For example, recent position papers highlight the role of agents such as vericiguat in patients with worsening HF despite optimized therapy, suggesting that objective congestion assessment may contribute to identifying candidates for such therapies and refining risk stratification in clinical practice [38].

Patients with constrictive pericarditis may exhibit abnormal VExUS profiles reflecting severe venous congestion. However, similar venous Doppler abnormalities may also occur in other conditions associated with elevated right-sided pressures, such as severe tricuspid regurgitation or advanced right heart failure, while restrictive cardiomyopathy may present with elevated filling pressures but less pronounced respiratory variation. Consequently, VExUS findings should be interpreted in conjunction with established echocardiographic criteria for constrictive pericarditis, such as the Mayo Clinic criteria, which integrate ventricular interdependence (septal shift), annulus reversus, and prominent expiratory diastolic flow reversal in the hepatic veins. Beyond diagnostic considerations, isolated case reports suggest that VExUS may also help guide decongestive therapy and monitor treatment response in patients with constrictive pericarditis and systemic venous congestion, although dedicated studies are still lacking [39,40].

#### 5.3.5. Integration of VExUS with Lung Ultrasound and Clinical Assessment

A frequently raised question concerns how VExUS fits into the broader assessment of congestion in HF alongside lung ultrasound (LUS) and physical examination. Emerging consensus indicates that VExUS and LUS are complementary rather than interchangeable, as they interrogate different compartments of congestion. LUS directly detects pulmonary congestion through B-lines and pleural effusions, reflecting elevated left-sided filling pressures. In contrast, VExUS assesses systemic venous congestion by evaluating the IVC and venous Doppler patterns in the hepatic, portal, and renal veins, which reflect elevated right atrial and central venous pressures. Neither modality excludes the other; instead, together they provide a more comprehensive picture of volume status. Traditional clinical assessment (jugular venous distension, peripheral edema, pulmonary crackles, congestive hepatomegaly) remains fundamental, but integration of LUS and VExUS allows more objective and sensitive quantification of congestion, addressing the limitations of physical examination alone [41].

In clinical practice, discordant congestion patterns are frequently encountered and can only be recognized through an integrated approach. For example, a patient with numerous B-lines on LUS but a normal VExUS score likely has predominantly pulmonary (left-sided) congestion, with elevated left ventricular filling pressures but without a proportional rise in CVP. Conversely, a patient with severe VExUS abnormalities and minimal pulmonary B-lines may exhibit a phenotype dominated by right-sided or splanchnic congestion, such as isolated right HF or advanced chronic HF with predominant systemic fluid accumulation. These discordant LUS/VExUS patterns suggest distinct pathophysiological phenotypes that may require tailored therapeutic strategies [42]. Such patterns may also provide additional insights when evaluating patients presenting with acute HF, potentially helping to differentiate de novo acute HF from acute decompensation of chronic HF.

Current literature suggests interpreting VExUS findings in conjunction with clinical data, LUS and echocardiography to achieve a holistic assessment of congestion. This multimodal approach may enhance diagnostic accuracy and may better guide therapeutic decisions, ultimately supporting more personalized management of patients with HF. Within this multimodal approach, VExUS may complement implantable hemodynamic monitoring systems by capturing the systemic venous consequences of elevated right-sided pressures, and could represent a practical non-invasive alternative in settings where invasive monitoring is unavailable. Moreover, VExUS-derived congestion phenotypes may help refine clinical decision-making, including the management of diuretic resistance, reassessment of cardiac resynchronization therapy response, or consideration of advanced HF therapies. However, these potential applications require validation in prospective studies.

Substantial evidence accumulated between 2018 and 2025 supports the utility of VExUS as a non-invasive tool for quantifying systemic venous congestion in HF and beyond. Foundational studies in intensive care established the conceptual framework by linking systemic venous congestion to postoperative AKI [6], while subsequent investigations in HF expanded its clinical applicability. VExUS has been shown to correlate with both right-and left-sided filling pressures [20], demonstrate prognostic value in predicting hospital readmissions and mortality [21], and offer potential guidance for tailoring decongestive therapies [43]. Importantly, it has become clear that VExUS does not replace, but rather complements other modalities of congestion assessment, including lung ultrasound, clinical examination, natriuretic peptides, and invasive hemodynamic monitoring. By integrating these approaches, VExUS enables a more nuanced assessment of fluid balance and volume tolerance on an individualized basis, having the potential of being a useful tool in Cardiology practice.

Despite certain limitations, including operator dependence and training requirements, venous congestion imaging may represent a valuable adjunct tool in cardiology practice across multiple stages of care: at admission, during hospitalization, at discharge, and during outpatient follow-up, as summarized in Table 2. Also, the potential role of VExUS in differentiating between various cardiovascular conditions associated with systemic venous congestion (such as isolated left heart failure, isolated right heart failure, right heart failure secondary to left heart failure, failed Fontan physiology, restrictive cardiomyopathy, arrhythmogenic right ventricular dysplasia) represents an additional area deserving further investigation.

## 6. Future Perspectives

As a novel hemodynamic tool, VExUS shows promise but requires rigorous validation. Current evidence derives largely from small cohorts and proof-of-concept studies. For example, single-center prospective series have linked high VExUS grades with mortality and rehospitalization in acute HF. However, multicentre, large-scale prospective cohorts are needed to establish its diagnostic thresholds and prognostic significance in diverse HF and chronic cardiorenal populations.

### 6.1. Integration with Other POCUS Modalities and Biomarkers

VExUS should not be used in isolation but as part of a multi-modal congestion assessment. Integrating LUS for pulmonary edema, focused echocardiography for cardiac function, and biomarkers could enhance risk stratification. For example, combining VExUS with NT-pro BNP (a marker of wall stress) may improve prediction of AKI and outcomes. Novel congestion biomarkers (such as CA125) should be explored alongside VExUS. HF patients with VExUS ≥ 2 had higher CA125 and microalbuminuria and an ~2.5-fold greater 1-year mortality risk [3]. This implies that combining VExUS ultrasound findings with serum markers could refine risk models. Furthermore, lung ultrasound B-lines (reflecting extravascular lung water) could complement VExUS: early work suggests a strong interplay between LUS findings and splanchnic congestion [41]. Future research should thus develop multiparametric protocols and test whether such integrated algorithms better predict outcomes or guide therapy than any single modality.

In this context, future studies could develop structured, algorithm-based approaches integrating VExUS with clinical assessment, laboratory markers, lung ultrasound and echocardiography to improve congestion phenotyping and guide therapeutic decision-making in HF.

### 6.2. Risk Stratification and Discharge Planning

VExUS has the potential to augment clinical decision-making at discharge and to contribute to risk stratification models. Small studies indicate high VExUS grades portend worse outcomes. In acute HF, patients presenting to the emergency department (ED) with VExUS grade 3 had significantly higher in-hospital mortality and early readmissions, whereas no patients with VExUS under 3 died [22]. Likewise, higher VExUS grades in the ED predicted 30-day mortality, supporting use of VExUS for risk stratification [44].

These findings raise the possibility of using VExUS at hospital discharge. In one study, the investigators prospectively measured VExUS just before discharge and found that HF patients with VExUS ≥ 2 had a markedly higher 90-day readmission/ED visit rate than those with VExUS 0 (35% vs. 9%, *p* = 0.044) [21]. This suggests that residual congestion on Doppler signals unmet by clinical exam portends post-discharge relapse. Future work should validate VExUS as part of discharge planning: for example, integrating VExUS score into risk models alongside NT-pro BNP. If patients with high discharge VExUS are confirmed at high risk, protocols could be tested (e.g., intensified diuresis, closer outpatient follow-up) to see if outcomes improve. In summary, VExUS could be incorporated into multi-parameter risk scores and transitional care plans, but this requires prospective validation in larger HF cohorts.

### 6.3. Automated and AI-Enhanced VExUS Scoring

Automation of VExUS interpretation is a promising frontier. AI algorithms could classify Doppler waveforms and compute congestion grades, reducing operator dependency. In a recent study, the AI model demonstrated high performance in classifying VExUS waveform patterns acquired by providers with varied experience levels. The overall accuracy across 300 waveforms was 97.3%, with high levels of accuracy for the evaluation of each specific vein Doppler pattern [45]. In other areas of ultrasound, deep learning has revolutionized image analysis; one recent review highlights that AI can automate image interpretation and provide decision support, potentially reducing clinician workload [46].

### 6.4. Training, Standardization, and Reproducibility

Ensuring consistent VExUS assessments across operators is critical. Early data suggest that VExUS can be reproducible, but only with proper technique. However, operator skill clearly influences accuracy [47]. Formal training protocols, standardized scanning procedures, and certification programs are needed. Simulated or web-based training modules (for Doppler physics and waveforms) could be developed and their impact studied. In parallel, consensus guidelines should define image acquisition standards (e.g., probe frequency, ECG gating, patient position) and scoring criteria.

### 6.5. Role in Advanced Heart Failure Therapies

The potential application of VExUS in patients undergoing advanced HF therapies, such as ventricular assist device implantation or heart transplantation, warrants further investigation. In these settings, accurate assessment of systemic venous congestion and right heart function is critical for perioperative risk stratification and postoperative management. Although venous Doppler abnormalities may theoretically provide complementary information regarding right-sided hemodynamic, particularly for predicting right heart failure after LVAD implantation or monitoring congestion following transplantation, dedicated studies evaluating the role of VExUS in these scenarios are currently lacking.

Similarly, VExUS may represent a useful adjunct for assessing systemic venous congestion in patients undergoing cardiac implantable electronic device (CIED) implantation. This population often includes patients with advanced age and multiple comorbidities, including HF, which increase the risk of device-related complications [48]. In this context, systemic venous congestion (detectable using the VExUS score) may influence the hemodynamic environment surrounding transvenous leads, potentially promoting endothelial injury, microthrombus formation, and bacterial adhesion, a hypothesis that warrants further investigation.

## 7. Limitations of the VExUS Score in Heart Failure

Despite its promise as a congestion assessment tool, VExUS score has several important limitations in the HF setting.

- Evidence Gaps: First, robust clinical trial evidence is lacking. The current literature is dominated by observational studies with relatively small sample sizes, with a notable absence of large randomized controlled trials validating VExUS-guided management in HF. Most data derive from acute or hospitalized cohorts, and its utility in stable chronic HF remains uncertain due to sparse studies in ambulatory populations.
- Technical and Operator Dependence: Second, VExUS is technically demanding and operator-dependent. Accurate Doppler waveform interpretation requires skill and experience, leading to potential inter-observer variability. Performing a full VExUS exam necessitates advanced ultrasound and Doppler expertise, and limited training in these techniques among general practitioners can impede reliable application [49]. Adequate visualization of hepatic and intrarenal venous Doppler signals may be challenging in some patients due to anatomical or patient-related factors such as obesity, bowel gas, limited acoustic windows, or suboptimal patient positioning, while accurate Doppler assessment may also be influenced by angle dependency and difficulties in aligning the Doppler beam with venous flow.
- Lack of Guideline Integration: Despite growing interest in venous Doppler–based congestion assessment, recent major guidelines such as the 2023 ESC Heart Failure update and the 2024 HFSA statements do not yet endorse routine VExUS use in standard HF care, thus the need for more validation. This likely reflects the fact that most available evidence derives from observational studies with relatively small cohorts, primarily focusing on diagnostic or prognostic associations rather than therapeutic guidance. In particular, randomized controlled trials evaluating whether VExUS-guided decongestive strategies improve clinically relevant outcomes, such as rehospitalization, renal function, or mortality, are currently lacking. Future prospective studies specifically designed to test VExUS-guided management algorithms will therefore be essential to determine its clinical utility and to support potential integration into guideline-directed HF management.
- Physiological and methodological confounders that may affect the interpretation of venous Doppler patterns: Severe tricuspid regurgitation represents an important limitation, as retrograde transmission of right-atrial systolic pressure waves can produce hepatic vein systolic flow reversal independently of true systemic venous congestion, potentially leading to overestimation of congestion severity.
  Similarly, right ventricular dilation or systolic dysfunction may modify venous Doppler morphology through alterations in right-atrial pressure dynamics and ventricular–atrial coupling, thereby influencing hepatic and portal venous flow patterns independent of intravascular volume status.
  Cardiac rhythm disturbances, particularly atrial fibrillation, may further complicate waveform interpretation because the loss of coordinated atrial contraction alters the physiological components of hepatic venous flow and contributes to increased beat-to-beat variability.
  In addition, the IVC diameter and collapsibility may be influenced by several patient-specific factors (obesity, high athletic conditioning, mechanical ventilation, or elevated intra-abdominal pressure), conditions in which standard IVC cut-off values may not reliably reflect right atrial pressure. These considerations emphasize that VExUS findings should be interpreted within the broader hemodynamic and clinical context rather than used as a standalone marker of congestion [4,6,50].
- Underlying liver disease is a potential confounder: because VExUS incorporates hepatic and portal venous Doppler patterns, these parameters may also be influenced by intrinsic hepatic pathology such as metabolic-associated fatty liver disease or liver cirrhosis. Portal hypertension, increased intrahepatic vascular resistance, and reduced hepatic compliance may alter portal and hepatic venous waveforms independently of cardiac-related venous congestion, potentially limiting the specificity of VExUS-derived parameters in this population. Therefore, VExUS findings in patients with concomitant liver disease should be interpreted cautiously and integrated with clinical context, echocardiographic evaluation, and other markers of congestion [50,51].

Taken together, these limitations underscore that while VExUS shows prognostic and diagnostic potential, further prospective trials and standardization are needed before it can be universally recommended or integrated into standard HF care. This calls for continued research to determine whether VExUS-guided therapy can improve outcomes and to clarify its role in chronic HF management.

## 8. Conclusions

VExUS has emerged as a valuable non-invasive tool for quantifying systemic venous congestion and its clinical impact, with evidence from critical care and cardiology settings indicating that high grades identify patients with significant fluid overload and predict adverse outcomes. This promise is grounded in the underlying pathophysiology: systemic venous congestion reflects true fluid overload and cardiorenal burden. Sustained elevation of venous pressures transmits to end-organs (kidneys, liver, gut), impairing perfusion and promoting organ dysfunction. Looking forward, it has the potential to personalize HF management by directly determining the congestion phenotype, and it can help tailor decongestive treatments to the individual patient’s needs. In practice, combining VExUS with lung ultrasound, echocardiography, biomarkers and clinical data can yield a nuanced profile of volume status and cardiac function; such multimodal algorithms may enable earlier identification of patients at high risk, guide titration of therapy to the patient’s tolerance, and ultimately improve outcomes through a more precise, patient-specific approach to volume management. In summary, as research and standardization progress, VExUS is poised to become an important component of personalized care in both critical illness and heart failure.

## Figures and Tables

**Table 1:** Key clinical studies evaluating the VExUS score and ultrasound markers of systemic venous congestion in heart failure.

Study	Population	N	Study Design	Key Findings
**Beaubien-Souligny et al., 2020 **[6]	Non-critically ill cardiac surgery patients	145	Prospective observational	Development of the VExUS grading system; higher grades associated with post-operative AKI
**Torres-Arrese et al., 2023 **[5]	Acute heart failure patients	74	Prospective multicentric	Venous congestion patterns associated with worse outcomes in HF
**Anastasiou et al., 2024 **[20]	Acute heart failure patients	200	Prospective observational	Multiorgan congestion assessed by VExUS associated with adverse outcomes (in-hospital mortality)
**Rinaldi et al., 2024 **[21]	Acute decompensated HF (ADHF) patients	49	Prospective observational	Elevated VExUS at discharge predicted readmission due to ADHF
**Landi et al., 2024** [22]	Emergency department HF patients	50	Prospective observational	Higher VExUS scores associated with worse outcomes
**Longino et al., 2023** [23]	Patients undergoing right heart catheterization	51	Prospective observational	VExUS predicted elevated right atrial pressure with high diagnostic accuracy
**Campos Sáenz de Santamaría et al., 2025** [3]	Hospitalized acute HF patients	100	Prospective observational	Multimodal congestion assessment including VExUS associated with outcomes
**Saadi et al., 2025** [4]	Acute decompensated HF	104	Prospective observational	Modified VExUS score predicted mortality
**Hassan et al., 2025** [24]	Ambulatory HFrEF patients	109	Prospective exploratory	VExUS ≥ 2 predicted mortality and HF hospitalization
**Abu-Naeima et al., 2025** [25]	Acute cardiorenal syndrome	43	Prospective cohort	Higher VExUS associated with reduced diuretic efficiency
**Alday-Ramírez et al., 2025** [26]	Pre-capillary pulmonary hypertension	49	Prospective	VExUS correlated with right atrial pressure

**Table 2:** Potential Integration of the VEXUS Score in Cardiology Practice.

Stage of Care	Application of VEXUS Score & Key Potential Clinical Implications
**At Admission**	**Objective:** Early detection of severe systemic venous congestion upon hospital presentation. **Findings:** Patients with VExUS grade 3 at initial ED evaluation had significantly higher in-hospital mortality and early readmissions, whereas no patients with VExUS under 3 died [22].
	**Implications:** Flags the need for prompt and efficient decongestive therapy (e.g., high-dose diuretics or ultrafiltration) to relieve congestion. Early VExUS assessment thus identifies high-risk patients on arrival who may benefit from intensified initial therapy.
**During** **Hospitalization**	**Objective:** Guide and titrate decongestive therapy through serial congestion assessments. **Assessment:** Repeated VExUS measurements during the inpatient stay indicate the current level of systemic venous congestion. A persistently high VExUS score signifies ongoing congestion and insufficient decongestion [3]. Conversely, improvement or normalization of VExUS parameters signifies effective decongestion, a signal to stop further aggressive diuresis to avoid adverse effects.
	**Implications:** VExUS provides real-time feedback to achieve a delicate balance in fluid management. While large trials are pending, early evidence suggests that integrating VExUS (or its components) into daily rounds helps ensure congestion is fully relieved avoiding over diuresis.
**At Discharge**	**Objective:** Confirm resolution of systemic congestion before hospital discharge. **Findings:** Residual venous congestion at discharge is a strong predictor of early relapse in HF patients. Patients discharged with an abnormal VExUS grade (≥2, indicating ongoing congestion) have significantly higher short-term readmission rates. In one cohort, ~35% of patients with VExUS grade 2–3 at discharge experienced HF readmission or urgent visits within 90 days, versus ~9% of those with a normal VExUS (grade 0) [21].
	**Implications:** A persistently elevated VExUS score at discharge identifies patients who have residual congestion despite clinical optimization. Integrating a VExUS evaluation into the pre-discharge workup ensures that decongestive therapy has been sufficient. The goal is to achieve a low VExUS grade (minimal venous congestion) by discharge, which is crucial for improving post-discharge prognosis and reducing the risk of early HF readmissions.
**Outpatient Monitoring**	**Objective:** Early detection of re-accumulating congestion in high-risk ambulatory patients. **Usage:** Periodic VExUS assessments during follow-up visits could uncover subtle volume overload before a patient becomes overtly decompensated. A rising VExUS score in the outpatient setting may indicate insidious fluid accumulation even if the patient appears clinically stable.
	**Implications:** a rising VExUS score may indicate insidious fluid accumulation, allowing clinicians to intervene (for example, by adjusting diuretic dose or lifestyle factors) before congestion leads to hospitalization. There is a need for more data in this regard.

## Data Availability

No new data were created or analyzed in this study. Data sharing is not applicable to this article.
